# Polyunsaturated fatty acid in treatment resistant depression: A pilot study

**DOI:** 10.1192/j.eurpsy.2021.868

**Published:** 2021-08-13

**Authors:** T. Jannini, L. Longo, Y. Barone, C. Niolu, S. Bernardini, A. Siracusano, P. Bertucci, G. Di Lorenzo

**Affiliations:** Department Of Systems Medicine, University of Rome Tor Vergata, Rome, Italy

**Keywords:** fatty acids, Treatment Resistant Depression, omega3, omega6

## Abstract

**Introduction:**

The deficiency of polyunsaturated fatty acids (PUFAs) and an alteration between the ratio of omega-6 and omega-3 PUFAs may contribute to the pathogenesis of depressive disorders.

**Objectives:**

To investigate the levels of omega-3 and omega-6 in red cell membranes (mPUFAs) and plasma (pPUFAs) of patients with treatment-resistant (TRD) and non-treatment resistant depression (non-TRD).

**Methods:**

TRD and non-TRD consisted of 75 patients enrolled at the Psychiatric and Clinic Psychology Unit of the University of Rome Tor Vergata, Rome, Italy, and met the DSM-IV criteria for major depressive disorder (MDD). A group of healthy controls (HC) matched for agender and age was enrolled. All blood samples were performed in conditions of an empty stomach between 07:00 am and 09:00 am. For each subject were obtained 5 ml of whole blood with the use of tubes for plasma with EDTA as an anticoagulant. Eicosapentaenoic acid (EPA) and docosahexaenoic acid (DHA) for omega-3 and arachidonic acid (AA) for omega-6 were measured.

**Results:**

Levels of pPUFAs did not differ between the three groups. The mPUFAs were altered in the MDD. TRD and non-TRD had lower EPA and AA values respect to the HC. DHA in red cell membranes was lower in TRD than non-TRD and HC.

**Conclusions:**

Changes in levels of PUFAs in red cell membranes, but not in plasma, may be an important factor to evaluate the resistance to the pharmacological treatment.

